# Impact of a 6-Month Micronutrient-Dense Plant-Rich Nutrition Intervention on Health and Well-Being at the Worksite

**DOI:** 10.1155/2019/2609516

**Published:** 2019-04-30

**Authors:** Jay T. Sutliffe, Julia C. Gardner, Michelle M. Gorman, Mary Jo Carnot, Wendy S. Wetzel, Tricia Fortin, Chloe A. Sutliffe, Alison Adams

**Affiliations:** ^1^PRANDIAL Lab: Department of Health Sciences, Northern Arizona University, Flagstaff, AZ 86011, USA; ^2^Lifepath, Northern Arizona Healthcare, Flagstaff, AZ 86001, USA; ^3^Department of Psychological Sciences, Chadron State College, Chadron, NE, USA; ^4^Employee Assistance and Wellness, Northern Arizona University, Flagstaff, AZ 86011, USA; ^5^Department of Biology, Northern Arizona University, Flagstaff, AZ 86011, USA

## Abstract

This nonrandomized pilot study utilized the health belief model and the theory of planned behavior to assess the effectiveness of perceived behavioral control to determine the impact of a micronutrient-dense plant-rich (mNDPR) dietary intervention on employee health and wellness at the worksite. Seventy-one employees and/or spouses (≥18 years) who met the inclusion criteria were recruited from a regional medical center and a local university. Participants were provided more than 14 hours of in-person lecture combined with take-home materials, and electronic resources to support participants in their transition and adherence to the dietary plan. The study consisted of a 6-hour introductory session followed by weekly 1-hour meetings for 7 consecutive weeks and then monthly 1-hour meetings, for 4 consecutive months over the span of 6 months. Retention of participants was approximately 55 percent. Participants were assessed for measures of weight, waist circumference, and blood pressure; physiological measures of blood cholesterol, triglycerides, blood glucose, and hemoglobin A1c; and well-being measures of gastroesophageal reflux disease, depression, sleep, pain, and worksite productivity, pre-, mid-, and post-intervention. A significant reduction was seen in weight (*F*(2, 78) = 19.81, *p* < 0.001) with a mean reduction of 6.65 lb., waist circumference (*F*(2, 72) = 40.914, *p* < 0.001) with a mean reduction of 2.8 inches, total cholesterol (*F*(2, 70) = 19.09, *p* < 0.001) with a mean reduction of 17.81 mg/dL, HDL (*F*(2, 70) = 4.005, *p*=0.023) with a mean reduction of 3.61 mg/dL, LDL (*F*(2, 56) = 10.087, *p* < 0.001) with a mean reduction of 13.1 mg/dL, blood glucose (*F*(2, 70) = 6.995, *p*=0.002) with a mean reduction of 3.7 mg/dL, hemoglobin A1c (paired samples t (39) = 2.689, *p*=0.01) with a mean reduction of 0.118%, GERD (*F*(2, 72) = 7.940, *p*=0.001, MSE = 4.225) with a mean reduction of 1.4, depressive symptoms as measured by the PHQ 9 (*F*(2, 72) = 10.062, *p* < 0.001, MSE = 5.174) with a mean reduction of 2.0, and an improvement in sleep quality was seen as measured by the PSQI (*F*(2, 74) = 11.047, *p* < 0.001, MSE = 2.269) with a mean improvement of 1.3. In most cases, improvement occurred across the first two time periods and then leveled off. Blood pressure, triglycerides, pain measurements, and WPAI did not change over time. Effect sizes for significant pairwise comparisons indicated medium to large effects of practical significance. This intervention was therefore effective at improving employee health and well-being. Widespread worksite implementation should be considered to improve the overall wellness of employees.

## 1. Introduction

In 2014, more than 2.1 billion people, nearly 30% of the global population, were overweight or obese, and 5% of the deaths worldwide were attributable to obesity [1]. This presents a tremendous threat to human health as obesity is linked to numerous chronic diseases, including type 2 diabetes mellitus, cardiovascular disease, and certain cancers. Moreover, obesity and associated diseases are extremely costly; in 2014, it is estimated that 2.8% of the global gross domestic product (GDP) was spent on obesity-related diseases [1].

Change in dietary practices is an essential component of behavioral alteration that is required to address overweight and obesity. As individuals typically spend 40 hours/week or more at work, many decisions concerning dietary habits are implemented in the worksite. Worksite dietary interventions are often encouraged by employers, as improved nutrition results in better health, with the potential for concomitant improvement in presenteeism, reduced healthcare costs, and increased worksite productivity [2, 3]. Moreover, employers may be in a position to offer incentives for participation in such interventions, leading to improved participation in a program [4].

We have developed a nutrition intervention program that emphasizes the role of micronutrients in the diet. These are commonly overlooked, yet an essential component of nutrition. First, micronutrients are required as cofactors for a wide diversity of cellular processes, and plant foods that are rich in antioxidants, vitamins, minerals, and phytochemicals are strongly associated with reducing diabetes mellitus, cardiovascular, and obesity-related risk factors [5]. Second, there is evidence that foods low in micronutrients stimulate overeating and food cravings. Consequently, interventions focusing on macronutrients are frequently barriers to sustained weight loss and thus reduced the risk of obesity-related diseases; conversely, foods that are rich in micronutrients lead to satiety and thus reduce overeating [6].

We have previously conducted pilot studies with employees of Northern Arizona University and Northern Arizona Healthcare, involving 6, 9, or 12-week micronutrient-dense plant-rich (mNDPR) dietary interventions, involving education and weekly meetings, and have found significant improvements in risk factors for diabetes mellitus and cardiovascular disease, in addition to depressive symptoms [7–9] in all three studies. Here, we extend these studies to determine the longer-term effectiveness of a similar intervention involving weekly meetings for 8 weeks, followed by monthly meetings for an additional four months.

## 2. Materials and Methods

### 2.1. Study Design

The current study focused on how the overall health and wellness of working adult employees at two worksites in Arizona (Northern Arizona University (NAU) and Northern Arizona Healthcare (NAH)) were impacted by 6 months of nutrition education. The study consisted of 6 hours of introductory education (a video of speakers at a previous immersion event was provided online, to replace a planned in-person immersion event that was canceled because of extreme weather conditions), followed by weekly 1-hour meeting for 7 consecutive weeks, and then monthly 1-hour meeting for 4 consecutive months over the span of 6 months. The educational sessions discussed implementation of a mNDPR diet style and other lifestyle behaviors. Time 1 premeasurements were collected prior to the start of the nutrition education sessions during study week 0, Time 2 midmeasurements were collected at study week 8, and Time 3 postmeasurements were collected during study week 26. Pre-, mid-, and post-intervention wellness factors were measured by the following self-reported survey tools: Gastroesophageal Reflux Disease Questionnaire (GERDQ) [10], Pain Quality Assessment Scale (PQAS) [11], Patient Health Questionnaire-9 (PHQ 9) [12], Pittsburgh Sleep Quality Index (PSQI) [13], and Work Productivity and Activity Impairment (WPAI) [14]. In addition, pre-, mid-, and post-intervention anthropometric and blood-lipid assessment were made to determine systolic blood pressure (SBP), diastolic blood pressure (DBP), height, weight, and waist and hip circumference, body mass index (BMI), cholesterol, triglycerides, HbA1c, and glucose. To assist participants in self-evaluation of compliance, an electronic “food tracker” was used, allowing participants to self-report weekly intake of greens, colorful vegetables, fruits, nuts/seeds, beans, whole grains, fats, and meat/animal products. The number of times the participants entered data in the food tracker was also used as a measure of participation in the program. Similarly, attendance at the weekly/monthly meetings was recorded at each meeting.

This intervention was centered upon the health belief model that individuals will take action when they believe they can successfully avoid or alter a negative health condition [15]. In other words, when an individual feels they have the capability to influence a positive health outcome, they are more likely to alter their personal behavior. In an effort to provide a base for long-term adherence, we also introduced components from the theory of planned behavior to assess the effectiveness of perceived behavioral control [16].

### 2.2. Participants and Recruitment

Individuals who met the following criteria were invited to participate: employee, spouse, or adult dependent of an employee at NAU or NAH; 18–80 years of age; self-reported body mass index (BMI) of 28 or greater; self-reported waist circumference ≥35″ for females and ≥40″ for males; ready and willing to make a lifestyle change; not currently participating in a weight-loss program; and not taking any medications that could increase medical risk or that had weight loss as a primary side effect. Participants were recruited through electronic messaging, fliers, and website promotion by the Northern Arizona University (NAU) Department of Employee Assistance and Wellness (EAW) and Northern Arizona Healthcare (NAH) lifepath. The protocol and study design were approved by the NAH Institutional Review Board (IRB), and all participants provided written informed consent.

At the start of the program, there were a total of 71 participants, of whom 80.9 percent were female; 90.3 percent Caucasian, 8.5 percent Native American, 7 percent Hispanic, 2.8 percent Asian, and 1.4 percent others. The age ranged from 22 years to 69 years with the mean age of 47.5, SD 10.8, and a median age of 48 years. Of these, 94.4 percent were employees.

Of the 71 participants who started the study, approximately 54.9% completed the entire study, as determined by completion of most biometric and well-being measures at three time periods. This was a representative of most wellness variables, except for WPAI. For the weekly food trackers at 23 study weeks, 26.8 percent of participants completed all 23 measures. Only 6.9 percent attended all of the weekly/monthly meetings (10 time periods).

Participants did not receive financial compensation but employees were eligible for incentives through their worksite wellness program, potentially offsetting the cost of their personal health insurance premiums. For full credit of points, participants were required to participate in the entire study.

### 2.3. Instruments

Methods for determining outcome measures were described previously [7–9], including a medical history and demographic questionnaire, and standard methods for determining anthropometric measurements of height, weight, waist circumference, hip circumference, and blood pressure. Additional outcome measures used in the present study were gastroesophageal reflux disease (GERD), using the GERDQ; sleep quality, using the Pittsburgh Sleep Quality Index (PSQI); depressive symptoms, using the PHQ 9; work productivity and activity impairment, using the WPA1-GHA,WPA1-GHB,WPA1-GHC, and WPA1-GHD questionnaires; and pain, using the Pain Quality Assessment Scale (PQAS). Attendance at weekly/monthly meetings was used as a measure of participation in the program. Adherence was encouraged and measured by participants completing a weekly electronic self-reporting survey that recorded the food and meals consumed that adhered to this intervention's dietary guidelines—primarily leafy greens, colorful vegetables, fresh or frozen fruits, legumes, raw nuts or seeds, whole grains, fats/oils, as well as low intake of meat or animal products. This dietary tracking was intended as a self-check for participants, but completion of the tracker each week was also used as a measure of participation in the program. All surveys and questionnaires were collected electronically utilizing Research Electronic Data Capturing tool (REDCap) [17].

### 2.4. Measures

Anthropometric measures were weight, waist circumference, and blood pressure; well-being measures were gastroesophageal reflux disease, sleep, depression, pain, and worksite productivity; serum measures were blood cholesterol, triglycerides, glucose, and hemoglobin A1c. The blood-lipid profile was performed by trained technicians and conducted in which participants fasted for a minimum of 8 hours. The technicians utilized 35 ml capillary whole blood specimens obtained by finger stick applied to the CLIA-waived Alere Cholestech LDX System Analyzer (Alere, Abbott Rapid Diagnostics, Illinois, USA). All laboratory tests were conducted by Healthwaves (Healthwaves, Tempe, AZ, USA).

### 2.5. Procedures

Procedures were as described previously for 12-week [9] and 9-week [8] pilot interventions with the addition of the Pain Quality Assessment Scale (PQAS) [11] and extension of the intervention period to 6 months.

Our evidenced-based micronutrient-dense plant-rich (mNDPR) dietary protocol has been published earlier [7–9] and is designed to be (1) micronutrient rich (i.e., increased consumption of foods containing especially high levels of plant-derived phytochemicals, antioxidants, vitamins, and minerals); (2) nutritionally adequate and diverse; (3) hormonally favorable, avoiding carbohydrates with a high glycemic index that could elevate levels of serum insulin and minimizing animal protein that may invoke an inflammatory response; and (4) encouraging of regular intake, with an emphasis on meals and not snacks, with an overnight “fast” of at least 12 hours. In addition, our approach does not generally emphasize macronutrient percentages, portion sizes, or calorie counting. Our methodological approach was published earlier [7–9] and describes how the participants were provided with the acronym GBOMBS + T to provide a guide for a portion of their food selection. The acronym GBOMBS + T emphasizes the use of greens, beans, onions, mushrooms, berries, seeds and nuts, plus tomatoes. The use of a multivitamin containing B12, iodine, zinc, and vitamin D was also encouraged as well as the consumption of a relatively small amount of eicosapentaenoic docosahexaenoic acid from algae to assure consumption of comprehensive and adequate nutrients, given the small amount of animal products recommended by the program. Participants were encouraged to continue their current exercise habits with a goal of a minimum of 150 minutes of moderate intensity, physical activity per week. Participants were provided contact information for providers of health services at the worksite in the event that they needed those services.

### 2.6. Data Analysis

All data analysis procedures were performed using SYSTAT 13.2. One-way ANOVAs were used to assess changes over time at three measurement points for anthropometric measures, physiological measures, and well-being measures. Post hoc analyses using Tukey HSD with a criterion alpha of 0.05 were used to distinguish when there were significant differences between time periods. Since many of the dependent measures did not meet normality assumptions, Friedman's test for repeated measures was also used, followed by Wilcoxon pairwise comparisons. For HbA1C, measurements were made only at Time 1 and Time 3; thus, a paired samples *t* test was used.

## 3. Results

### 3.1. Anthropometric and Physiological Measures

As shown in Table 1, there were significant changes in weight across time (*F*(2, 78) = 19.81, *p* < 0.001). Post hoc analyses indicated that there was a reduction in weight between Times 1 and 2, but no change after that point (Time 1 was different from Times 2 and 3). These differences were confirmed in nonparametric testing due to nonnormality of sample data (Friedman test = 27.846, *p* < 0.001; Wilcoxon tests, *p* < 0.001).

There were also significant changes in waist measurement across time (*F*(2, 72) = 40.914, *p* < 0.001). Post hoc analyses indicated that there was a reduction in waist measurement between Time 1 and Time 2, but no change after that point. Nonparametric tests indicated the same conclusion. These differences were confirmed in nonparametric testing due to nonnormality of sample data (Friedman test = 34.900, *p* < 0.001; Wilcoxon tests, *p* < 0.001).

There were no significant changes in systolic blood pressure (*F*(2, 76) = 2.413, *p*=0.104) or diastolic blood pressure (*F*(2, 76) = 1.306, *p*=0.277) across the three time periods tested. This was confirmed in nonparametric tests: systolic blood pressure (Friedman = 2.849, *p*=0.241) and diastolic blood pressure (Friedman = 2.537, *p*=0.281).

There were significant changes in total cholesterol levels across time (*F*(2, 70) = 19.09, *p* < 0.001), in HDL (*F*(2, 70) = 4.005, *p*=0.023) and in LDL (*F*(2, 56) = 10.087, *p* < 0.001). Based on post hoc tests, there were reductions in total cholesterol, HDL and LDL between Times 1 and 2, but no change after that point. The cholesterol ratio did not change significantly over time (*F*(2, 66) = 0.121, *p*=0.887). Each of these differences was confirmed in nonparametric tests (total cholesterol: Friedman = 28.671, Wilcoxon *p* < 0.001; HDL: Friedman = 7.137, *p*=0.028, Wilcoxon *p* < 0.001; LDL: Friedman = 13.310, *p*=0.001, Wilcoxon *p* ≤ 0.001; cholesterol ratio: Friedman = 0.870, *p*=0.647).

There were significant changes in blood glucose levels across time (*F*(2, 70) = 6.995, *p*=0.002). Post hoc comparisons indicated that Time 1 was different from Time 2 but not from Time 3 and that Times 2 and 3 were not different from each other. There were no significant changes in triglycerides over time (*F*(2, 68) = 0.418, *p*=0.660). These differences were confirmed in nonparametric tests (glucose: Friedman = 18.142, *p* < 0.001. Wilcoxon *p* < 0.001; triglycerides: Friedman = 3.215, *p*=0.200).

There was a significant reduction in hemoglobin HbA1C between Time 1 and Time 3 (paired samples *t*(39) = 2.689, *p*=0.01). HbA1c levels at Time 2 were not measured because a minimum of 3 months is required in order to detect a change in HbA1c [18]. This was confirmed in the Wilcoxon test (*z* = −3.533, *p* < 0.001).

### 3.2. Well-Being Measures

As shown in Table 2, there was a significant change in GERD over time as measured by the GERDQ (*F*(2, 72) = 7.940, *p*=0.001, MSE = 4.225). Post hoc comparisons indicated there was a reduction in the GERD score from Time 1 to Times 2 and 3, but Times 2 and 3 did not differ (Friedman = 9.620, *p*=0.006, Wilcoxon *p* < 0.05).

Similarly, there was a significant reduction in depressive symptoms as measured by the PHQ 9 over time (*F*(2, 72) = 10.062, *p* < 0.001, MSE = 5.174). Post hoc comparisons indicated that Time 1 was different from Times 2 and 3, but Times 2 and 3 did not differ (Friedman = 9.887, *p*=0.007, Wilcoxon *p* ≤ 0.003).

There was also a significant change in sleep over time, as measured by the PSQI (*F*(2, 74) = 11.047, *p* < 0.001, MSE = 2.269). Post hoc comparisons indicated that the Time 1 PSQI score was higher (indicating more sleep difficulties) than Times 2 and 3, but Times 2 and 3 did not differ (Friedman 14.637, *p* < 0.001, Wilcoxon *p* ≤ 0.001).

With most of the pain measures (PQAS), there were no significant changes across the three time periods using parametric measures. However, nonparametric tests indicated a difference between Times 1 and 3 for total pain scores (PQAS-total: *F*(2, 72) = 1.662, *p*=0.197, MSE = 326.317 no significant difference, Friedman = 6.041, *p*=0.049; proximal: *F*(2, 72) = 0.743, *p*=0.479, MSE = 25.021 no significant difference, Friedman = 3.792, *p*=150; surface *F*(2, 72) = 0.896, *p*=0.413 MSE = 8.413, no significant difference, Friedman = 2.619, *p*=0.270: deep pain *F*(2, 72) = 3.108, *p*=0.051, MSE = 37.461, Friedman = 3.318, *p*=0.190). Analysis of worksite activity, impairment, and time missed due to health issues indicated there were no significant changes across time (WPAI_GHA: no significant changes across time *F*(2, 42) = 0.430, *p*=0.653, MSE = 0.013, Friedman = a.250, *p*=0.882; WPAI-GHB: no significant changes across time *F*(2, 76) = 1.284, *p*=0.283, MSE = 2.467, Friedman = 2.774, *p*=0.250; WPAI_GHC: no significant changes across time *F*(2, 42) = 0.272, *p*=0.764, MSE = 1.700, Friedman = 0.740, *p*=0.691; and WPAI_GHD: no significant changes across time *F*(2, 72) = 0.272, *p*=0.764, MSE = 2.668, Friedman = 0.705, *p*=0.703).

Pairwise mean changes and effect sizes are included in Table 2. Ivarsson et al. have suggested that both parametric and nonparametric effect sizes should be reported [19]. Cohen's *d* is the appropriate parametric measure of effect size for paired sample *t* tests. For Wilcoxon tests, *r* is a more appropriate measure for these paired comparisons. Fritz et al. provided a table comparing several measures of effect size and showing equivalencies [20]. Based on Cohen's conventions, Cohen's *d* = 0.20 is a small effect, Cohen's *d* = 0.50 is a medium effect, and Cohen's *d* = 0.80 is a large effect. According to Table 7 in the study by Fritz et al. [20], the equivalent values of *r* are 0.10 (small), 0.24 (medium), and 0.37 (large). Changes reported as significant in statistical tests in this study are most often between Time 1 and later time periods, with a leveling off between Times 1 and 2. Effect sizes are generally large, indicating practical significance of the results.

### 3.3. Attendance at Weekly Meetings, Completion of Food Trackers, and Participation in Outcome Measurements

**Figure 1:**
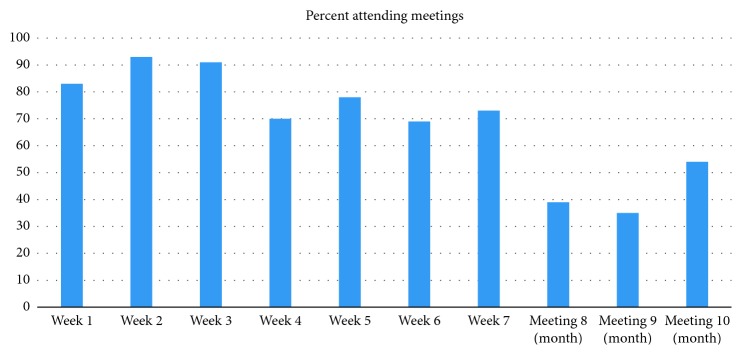
Percentage of participants attending meetings.

The average number of sessions attended was 6.9 (SD = 2.1) with the median number of sessions = 7 (Figure 1). Only 7 percent of individuals attended all 10 sessions. Attendance was better for the weekly sessions than for the monthly sessions. For the seven weekly sessions, the average number of sessions was 5.6 (SD = 1.4) with median of 6 sessions. On average, 80.3 percent of the weekly sessions were attended. For the three monthly sessions, the average number of sessions attended was 1.3 (SD = 1.1) with a median of 1 session. On average, 42.7 percent of the monthly sessions were attended.

**Figure 2:**
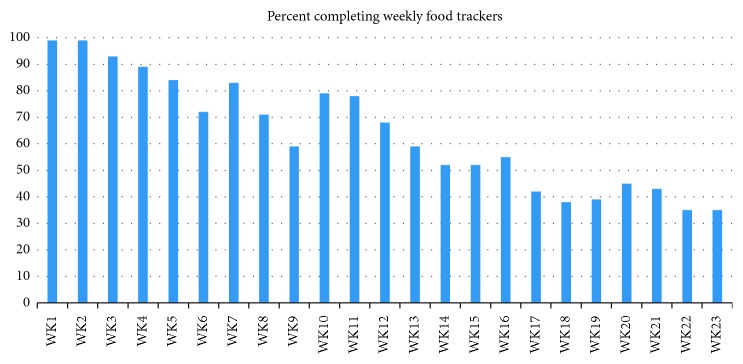
Percentage of participants completing weekly food trackers.

Participation in other aspects of the study also dropped off over time.  Figure 2 shows the percentage of participants at each time period who completed the weekly food trackers. During the study, there was a steady decline in the percentage of individuals completing the tracker.

**Figure 3:**
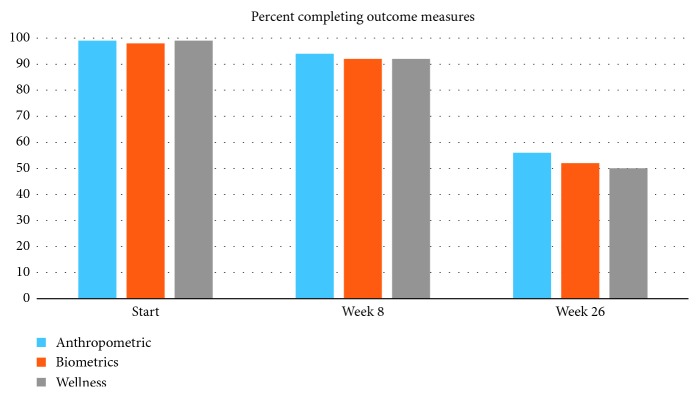
Percentage of participants completing outcome measures.

Participation in completing outcome measures fell only slightly from week 1 to week 8, but there was a large decrease in participation in the week 26 measures, as shown in Figure 3.

## 4. Discussion

In our previous studies, we showed that 6-, 9-, and 12-week mNDPR worksite interventions were all highly effective at improving anthropometric, biometric, and wellness measures [8, 9]. We did not have any information, however, as to the longer-term impact of these interventions and in particular as to whether participants were able to maintain the dietary changes and concomitant improvements in health associated with the various interventions. In the present study, we conducted an 8-week mNDPR intervention, using the same protocol as described previously, but followed the participants for the remaining 4 months. During the remaining 4 months, participants were provided with monthly newsletters and meetings that were intended to provide ongoing support, and continued participation in the program was monitored with the weekly food trackers, attendance at the monthly meetings, and at the end of 4 months with final anthropometric, biometric, and wellness measurements.

We found that, as described previously, in the first 8 weeks, there were highly significant improvements in weight, waist circumference, cholesterol, blood glucose, GERD, depressive symptoms, and sleep. Moreover, we found that by week 26, all these changes had been maintained, as there were no significant differences in any of these measures between the 8-week and 26-week measures. During the first 8 weeks, there were no significant changes in blood pressure, triglycerides, pain scores or worksite activity, impairment, and time missed due to health issues. By week 26, however, there was also a significant reduction in total pain, and measurement of HbA1c revealed a significant reduction compared with week 1. The finding that improvements in anthropometric, biometric, and wellness measures seen at the end of the intensive 8-week program were maintained for during the remaining 4 months is important and demonstrates that the program is sustainable for those that completed it, over at least a 6-month time period.

Participation in the program, however, showed significant reduction after the first 8 weeks (Figures 1 and 2). This was evident in (i) the number of participants who participated in the 26-week outcome measures (50–57% participation at week 26 compared with over 90% participation at weeks 1 and 8), (ii) the number of participants attending the meetings (a low of 35% at the penultimate monthly meeting) compared with a high of 94% at the second weekly meeting); and (iii) the number of participants filling in the weekly food trackers (there was a steady decline from 99 in week 1 to 37% in week 23). This finding is consistent with results from previous studies that show that intensity of programs is associated with success [21]. For more than 50% of individuals who completed the program, as judged by these measures, however, improvement in outcomes was maintained, despite a great reduction in participant contact and intensity of the program. As we do not have data on those who did not complete the 6-month outcome measurements, we have no way of knowing the 6-month success rate of these individuals; it is possible that some of these individuals were successful in participating in the mNDPR intervention and simply stopped participating in the study. For about 50% of the participants, however, highly significant improvements were seen over the 6-month period.

Further studies are needed to determine (i) to what extent the improvement seen at 6 months is maintained longer term and (ii) whether it is possible to develop a post-intervention program that increases the number of individuals who participate beyond the initial intensive 8 weeks of the program.

The finding that the mNDPR intervention is effective in promoting weight loss and improving other risk factors for diabetes mellitus is consistent with results from numerous other studies that seek to prevent the onset of diabetes mellitus [21]. In the present study, we also examined wellness outcomes and found highly (*p* < 0.001) significant improvement in sleep, depressive symptoms, and GERD. Currently, there are no dietary recommendations for depression, but use of a micronutrient-dense plant-rich diet for reducing depression is consistent with other studies that show an impact of whole, plant-based food on depression [22].

Limitations of the study are that (i) this was a pilot study and did not have a control arm. Comparison was made of outcome measures pre-intervention and post-intervention for each individual. (ii) Participation in the study fell (as judged by the food trackers, attendance at the meetings, and participation in outcome measures), particularly at the end of the 8-week intensive intervention; thus, outcome measures are available for only about 50% of the initial participants.

## 5. Conclusion

The mNDPR intervention is highly effective in promoting improvement in risk factors for diabetes mellitus and cardiovascular disease, as well as in impacting wellness outcomes, including sleep, depression, and GERD. Moreover, this protocol is effective as a worksite intervention. This type of intervention therefore appears to be not only beneficial for metabolic health but also have a broader impact on other wellness factors, such as depression and sleep. Each of these factors impacts the others; so, there are likely synergistic impacts of a mNDPR approach.

## Figures and Tables

**Table 1:** Mean and SD anthropometric and physiological measures of variables pre- and post-intervention.

Biometric measurement	Statistical significance	Time point	*n* ^*∗*^	Mean ± SD	CI, 95 %	Parametric	Nonparametric	
						Cohen's *d*	*r*	*z*
Weight (kg)	*F*(2, 78) = 19.81, *p* < 0.001, Time 1 different	1-2	40	6.6 ± 4.3	5.2, 7.9	1.51	−0.8	−5.2
		1–3	40	6.7 ± 9.2	2.7, 8.6	0.72	−0.5	−3.3
		2-3	40	−0.9 ± 6.9	−3.1, 1.3	0.13	0.1	0.6

Waist (cm)	*F*(2, 72) = 40.914, *p* < 0.001, Time 1 different	1-2	37	2.7 ± 2.0	2.0, 3.4	1.36	−0.8	−4.9
		1–3	37	2.8 ± 2.5	2.0, 3.7	1.11	−0.8	−4.6
		2-3	37	0.1 ± 1.8	−0.5, 0.7	0.06	−0.02	−0.1

Systolic blood pressure (mmHg)	*F*(2, 76) = 2.413, *p*=0.104, no change	1-2	39	4.0 ± 11.4	0.3, 7.7	0.35	−0.3	−2.0
		1–3	39	0.7 ± 13.3	−3.7, 5.0	0.05	−0.05	−0.3
		2-3	39	−3.3 ± 14.4	−8.0, 1.4	0.23	0.2	1.4

Diastolic blood pressure (mmHg)	*F*(2, 76) = 1.306, *p*=0.277, no change	1-2	39	2.3 ± 8.5	−0.5 < mu < 5.0	0.27	−0.3	−1.7
		1–3	39	1.5 ± 8.4	−1.3 < mu < 4.2	0.18	−0.2	−1.2
		2-3	39	−0.8 ± 9.9	−4.0 < mu < 2.404	0.08	0.08	0.5

Total cholesterol (mg/dL)	*F*(2, 70) = 19.09, *p* < 0.001, Time 1 different	1-2	36	20.1 ± 20.3	13.2 < mu < 26.9	0.99	−0.8	−4.7
		1–3	36	17.8 ± 24.2	9.6 < mu < 26.0	0.73	−0.6	−3.8
		2-3	36	−2.2 ± 19.1	−9.7 < mu < 4.2	0.12	0.1	0.6

HDL (mg/dL)	*F*(2, 70) = 4.005, *p*=0.023, Time 1 different	1-2	36	3.4 ± 9.0	0.4 < mu < 6.5	0.38	−0.5	−2.7
		1–3	36	3.6 ± 8.2	0.8 < mu < 6.4	0.44	−0.4	−2.4
		2-3	36	0.2 ± 8.8	−2.8 < mu < 3.1	0.02	0.1	0.7

LDL (mg/dL)	*F*(2, 56) = 10.087, *p* < 0.001, Time 1 different	1-2	29	14.1 ± 17.7	7.4, 20.9	0.8	−0.7	−3.6
		1–3	29	13.1 ± 21.3	5.0, 21.2	0.62	−0.6	−3.0
		2-3	29	−1.3 ± 17.5	−7.7, 5.6	0.07	0.1	0.5

Cholesterol: HDL ratio	*F*(2, 66) = 0.121, *p*=0.887, no change	1-2	34	0.1 ± 1.4	−0.4, 0.6	0.06	−0.2	−1.2
		1–3	34	0.1 ± 1.3	−0.4, 0.6	0.07	−0.2	−1.0
		2-3	34	0.01 ± 1.0	−0.4, 0.4	0.01	−0.01	−0.04

Glucose (mg/dL)	*F*(2, 70) = 6.995, *p*=0.002, Time 1 different	1-2	36	9.1 ± 10.6	5.5, 12.6	0.86	−0.7	−4.2
		1–3	36	3.7 ± 16.2	−1.8, 9.2	0.23	−0.3	−1.8
		2-3	36	−5.3 ± 16.3	−10.8, 0.2	0.33	0.4	2.2

Triglycerides (mg/dL)	*F*(2, 68) = 0.418, *p*=0.660, no change	1-2	35	9.7 ± 55.8	−9.5, 28.9	0.17	−0.2	−1.0
		1–3	35	−0.3 ± 78.1	−27.2, 26.5	0	0.1	0.6
		2-3	35	−10.0 ± 84.3	−39.0, 18.9	0.12	0.3	1.5

HbA1c (%)	Paired samples *t* (39) = 2.689, *p*=0.01	1–3	40	0.1 ± 0.3	0.1, 0.2	0.43	−0.6	−3.5

^*∗*^Values of *n* are for numbers of participants who completed biometric and wellness measures at all 3 time points.

**Table 2:** Mean and SD well-being measures of variables pre-, mid-, and post-intervention.

Wellness measurement	Statistical significance	Time point	*n* ^*∗*^	Mean ± SD	CI, 95%	Parametric	Nonparametric	
						Cohen's *d*	*r*	*z*
GERD (GERDQ)	*F*(2, 72) = 7.940, *p*=0.001, Time 1 different	1-2	37	2.1 ± 3.6	0.9, 3.3	0.58	−0.5	−3.1
		1–3	37	2.0 ± 3.7	0.8, 3.3	0.55	−0.5	−3.0
		2-3	37	−0.1 ± 2.1	−0.8, 0.7	0.03	0.03	0.2

PSQI (sleep)	*F*(2, 74) = 11.047, *p* < 0.001, Time 1 different	1-2	38	1.5 ± 2.2	0.8, 2.2	0.69	−0.6	−3.7
		1–3	38	1.3 ± 2.1	0.6, 2.0	0.61	−0.6	−3.4
		2-3	38	−0.2 ± 2.1	−0.9, 0.5	0.1	0.1	0.7

WPAI_A	*F*(2, 42) = 0.430, *p*=0.653, no change	1-2	22	0.001 ± 0.2	−0.1, 0.1	0.01	−0.1	−0.3
		1–3	22	−0.03 ± 0.2	−0.1, 0.1	0.16	0.1	0.5
		2-3	22	−0.03 ± 0.2	−0.1, 0.1	0.17	0.1	0.4

WPAI_B	*F*(2, 76) = 1.284, *p*=0.283, no change	1-2	39	0.4 ± 1.4	−03, 0.9	0.31	−0.3	−2.0
		1–3	39	0.5 ± 2.4	−0.3, 1.3	0.22	-0.2	−1.4
		2-3	39	0.1 ± 2.6	−0.8, 1.0	0.04	−0.1	−0.7

WPAI_C	*F*(2, 42) = 0.272, *p*=0.764, no change	1-2	22	0.3 ± 1.4	−0.3, 0.9	0.2	−0.2	−0.7
		1–3	22	0.1 ± 1.9	−0.8, 0.9	0.05	−0.1	−0.5
		2-3	22	−0.2 ± 2.2	−1.2, 0.8	0.09	0.1	0.5

WPAI_D	*F*(2, 72) = 0.272, *p*=0.76, no change	1-2	37	0.5 ± 1.8	−0.1, 1.0	0.26	−0.2	−1.0
		1–3	37	0.2 ± 2.6	−0.7, 2.6	0.07	−0.2	−1.2
		2-3	37	−0.3 ± 2.5	−1.1, 0.6	0.11	0.03	0.2

PHQ 9 (depressive symptoms)	*F*(2, 72) = 10.062, *p* < 0.001, time 1 different	1-2	37	2.1 ± 3.6	0.9, 3.3	0.58	−0.5	−3.1
		1–3	37	2.0 ± 3.7	0.8, 3.3	0.55	−0.5	−3.0
		2-3	37	−0.1 ± 2.1	−0.8, 0.7	0.03	0.03	0.2

PQAS total (pain)	*F*(2, 72) = 1.662, *p*=0.197, no change. Friedman significant	1-2	37	5.7 ± 24.9	−2.6, 14.1	0.23	−0.4	−2.2
		1–3	37	7.3 ± 25.0	−1.1, 15.6	0.29	−0.4	−2.5
		2-3	37	1.5 ± 26.6	−7.4, 10.4	0.06	−0.1	−0.6

PQAS proximal pain	*F*(2, 72) = 0.743, *p*=0.479, no change	1-2	37	0.3 ± 7.0	−2.1, 2.6	0.04	−0.1	−0.5
		1–3	37	1.3 ± 7.0	−1.0, 3.7	0.19	−0.3	−1.6
		2-3	37	1.1 ± 7.2	−1.3, 3.5	0.15	−0.2	−1.1

PQAS surface pain	*F*(2, 72) = 0.896, *p*=0.413, no change	1-2	37	0.3 ± 3.9	−1.0, 1.6	0.08	−0.1	−0.5
		1–3	37	0.9 ± 5.0	−0.8, 2.5	0.18	−0.2	−1.1
		2-3	37	0.6 ± 3.3	−0.5, 1.7	0.17	−0.3	−1.8

PQAS deep pain	*F*(2, 72) = 3.108, *p*=0.051, no change	1-2	37	3.5 ± 9.0	0.5, 6.5	0.39	−0.4	−2.1
		1–3	37	2.6 ± 8.2	−0.1, 5.4	0.32	−0.4	−2.2
		2-3	37	−0.8 ± 9.3	−4.0, 2.3	0.09	0.1	0.5

^*∗*^Values of *n* are for numbers of participants who completed biometric and wellness measures at all 3 time points. WPAI:GH A2 is the percent activity impairment due to health, WPAI:GH B2 is the percent impairment while working due to health, WPAI:GH C2 is the percent overall work impairment due to health, and WPAI:GH *D* is the percent work time missed due to health.

## Data Availability

The data used to support the findings of this study are available from the corresponding author upon request.
